# Comment on Clomiphene or enclomiphene citrate for the treatment of male hypogonadism: a systematic review and meta-analysis of randomized controlled trials

**DOI:** 10.20945/2359-4292-2025-0516

**Published:** 2025-11-22

**Authors:** Bo Peng

**Affiliations:** 1 Department of Center for Reproductive Medicine, The Quzhou Affiliated Hospital of Wenzhou Medical University, Quzhou People’s Hospital, Quzhou, Zhejiang, China

Dear Editor,

I carefully reviewed the authors’ systematic review and meta-analysis on clomiphene/enclomiphene (SERMs) for functional male hypogonadism (^1^). The work provides valuable pooled evidence for fertility-preserving scenarios. However, several issues merit discussion.

First, the included randomized-control trials (RCTs) span heterogeneous populations (obesity/metabolic disturbance, borderline low testosterone, variable symptom burden), dosing/regimens (clomiphene vs enclomiphene pooled), and short follow-up. While subgroup/meta-regression analyses address statistical heterogeneity, clinical heterogeneity is underexplored-particularly pharmacokinetic/pharmacodynamic and estradiol dynamics that may differ between the two agents, arguing for separate reporting to improve interpretability. Furthermore, functional secondary hypogonadism differs etiologically from organic primary hypogonadism, with better expected response to SERMs in the former (^2^). Conclusions and practice implications should more explicitly delimit the target population and avoid extrapolation to organic hypogonadism or discordant symptom-total testosterone presentations.

Second, biochemical outcomes (total testosterone, luteinizing hormone, follicle-stimulating hormone, estradiol) dominate, whereas patient-centered outcomes (libido/erectile function, fatigue, quality of life, mood) are inconsistent or sparse. Prior work shows biochemical correction does not reliably translate to symptomatic benefit (^3^). We suggest predefining symptom scales (e.g., International Index of Erectile Function, quantitative Androgen Deficiency in Aging Males), bone health markers, body composition, and metabolic phenotypes as co-primary/key secondary outcomes, coupled with minimum clinically important difference thresholds to prevent “biochemical difference-clinically trivial” interpretations.

Third, the analysis shows SERM superiority over testosterone gel for semen parameters-consistent with hypothalmaic-pituitary-gonadal-axis physiology-yet limited duration and sample size preclude confident inferences for pregnancy or live birth, the outcomes most meaningful to patients (^4^). Positioning SERMs, human chorionic gonadotropin, and combination strategies within modern male infertility care requires head-to-head trials powered for conception and live birth (^5^). The discussion should more cautiously grade the “fertility preservation” claim and prioritize future trials with reproductive hard endpoints.

In sum, this review supports SERM use in selected patients, but more granular guidance is needed on clinical stratification, patient-centered outcomes, and reproductive hard endpoints.

## Data Availability

datasets related to this article will be available upon request to the corresponding author.
